# Sample Preparation Protocol for Laboratory Cryo-Soft X-Ray Microscopy for Studying Cellular Nanoparticle Uptake

**DOI:** 10.3390/ijms26041657

**Published:** 2025-02-15

**Authors:** Komang G. Y. Arsana, Martin Svenda, Hans M. Hertz

**Affiliations:** Biomedical and X-Ray Physics, Department of Applied Physics, KTH Royal Institute of Technology, 10691 Stockholm, Swedenmartin.svenda@biox.kth.se (M.S.)

**Keywords:** water window, X-ray microscopy, cell imaging, cryofixation, sample preparation, nanoparticle

## Abstract

Soft X-ray microscopy (SXM) is a powerful technique for high-resolution biomedical imaging, enabling the observation of bio–nano interactions in near-native conditions without the need for heavy metal staining and fluorescence labeling. A laboratory soft X-ray microscope (LSXM) was developed to bridge the resolution gap between light microscopy and electron microscopy in cellular imaging. However, LSXMs employ a lower-brightness X-ray source in comparison to those operated in synchrotron facilities, which can negatively affect the contrast of X-ray micrographs. Therefore, proper sample preparation is essential to achieve optimal imaging results. This paper details an LSXM sample preparation protocol for investigating cellular nanoparticle uptake. Samples are prepared using optimized parameters for both manual plunge-freezing and automated vitrification, ensuring the rapid transition of biological material into a solid state with controllable thickness in the 5–10 μm range, preserving cellular structures and enabling optimal X-ray transmission for cellular imaging. We demonstrate the effectiveness of this protocol in facilitating the observation of nanoparticle uptake in two different biological samples: murine macrophages and acanthamoeba. Controlling ice thickness improves X-ray transmission through the specimen, enhancing the contrast and image quality of SXM.

## 1. Introduction

Soft X-ray microscopy (SXM) has emerged as an attractive imaging technique in cell biology. SXM allows the visualization of cellular architecture in its near-native state [1,2]. SXM has also seen increasing use in nanomedicine research, particularly in studies of nanoparticle cellular uptake through in vitro models [3,4,5,6]. This technique has been providing information on the ultra-structure of cells, complementing other imaging techniques such as optical microscopy and electron microscopy (EM) [7,8,9]. This is possible due to the natural contrast in the water window, which is a soft X-ray region with a wavelength between 2.3nm and 4.3nm or photon energy between 290eV and 540eV [10]. In this photon energy range, the X-ray photons are highly absorbed by carbon-based materials (e.g., proteins and lipids) and are less absorbed by water, allowing nanometer-resolution imaging of unstained biological samples up to 10 μm thick [2].

The soft X-ray microscopes used for studying the cellular mechanisms in response to nanoparticle exposure are mostly operated at synchrotron facilities. Such studies require an X-ray source with high spectral brightness. Currently, there are only a few beamlines at synchrotron facilities hosting soft X-ray microscopes for biological studies [11,12,13,14,15]. This, of course, limits the access of the scientific community to this technique. A laboratory soft X-ray microscope (LSXM) was developed in the early 2000s [16] as a complement to the synchrotron-based soft X-ray microscopes. This microscope has been proven to have comparable imaging capabilities [17,18]. However, the laboratory setups that are presently available have a lower-brightness X-ray source [19,20] and longer image acquisition times, which could negatively affect the image quality in cellular imaging.

In photon-limited imaging techniques such as with LSXMs, sample preparation is an important key factor in obtaining high-quality images and repeatable experimental results. The currently available protocols for cryofixed biological samples are actively being applied to either cryo-EM or synchrotron-based microscopy [21,22,23,24]. These protocols do not consider the importance of the amount of culture media or water around the cells of interest. It is also common to use coating and buffer solutions to increase cell adhesion. Both of these practices reduce the transmission of soft X-rays through the samples, resulting in a small number of photons reaching the detector. Consequently, they are less applicable for LSXMs.

In the present paper, we develop a detailed protocol for preparing biological samples to study the cellular uptake of metal-based nanoparticles (${\mathrm{MoO}}_{2}$ NPs). We fixed the samples by plunging them into liquid ethane using both a custom-designed plunge-freezing system and a commercially available automatic system. We demonstrate that controlling and monitoring the blotting parameters during plunge-freezing results in a 5–10 μm ice layer, the ideal thickness for studying bio–nano interactions. This optimized preparation protocol allows for the observation of the nanoparticle uptake and distribution within cells in their near-native cellular environment. Our approach provides reproducible results that improve the quality of the images acquired by LSXMs, highlighting the importance of optimized sample preparation for LSXMs in biological studies.

## 2. Results

### 2.1. Manual Plunge-Freezing System

Plunge-freezing is one of the most widely used cryofixation techniques in SXM due to its effectiveness and simplicity [25,26]. Manual plunge-freezing apparatuses, in particular, are economically feasible and can be easily modified to meet the specific requirements of a particular system. Although good results can be achieved with these systems, they often struggle to provide consistent outcomes within the same batches of samples.

**Figure 1:**
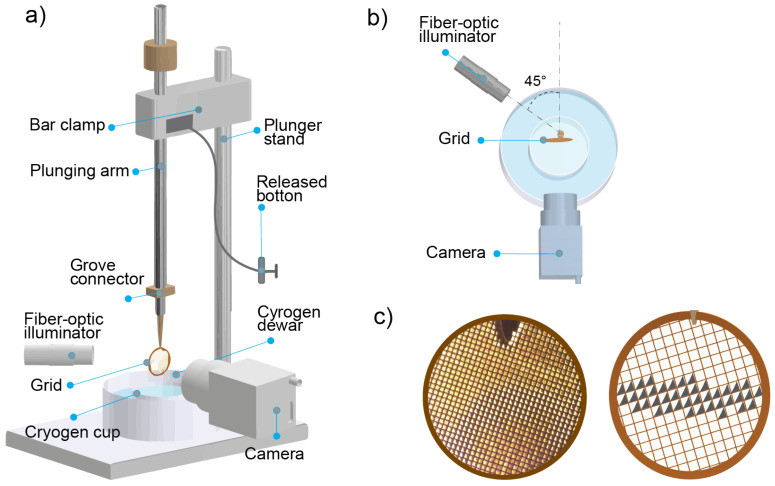
(**a**) Schematic of the manual plunge-freezing setup integrated with the monitoring system used in this protocol. Its main components are a stand, bar clamp, plunging arm, cryogen cup, cryogen dewar, and camera system. (**b**) The arrangement of the fiberoptic illuminator, grid, and camera seen from above. To be able to assess the water thickness, the illuminator has to be positioned at 45 degrees, as illustrated in the figure. (**c**) Image of TEM grids containing crescent-shaped patterns (**left**) and an illustration of its image (**right**).

Controlling the thickness of the water layer on biological samples during vitrification has long been a challenge in these systems [27]. This issue can be addressed by integrating a real-time monitoring system, as suggested in this study. We employed a manual plunge-freezer equipped with such a monitoring system, as illustrated in Figure 1a. The monitoring system consists of a camera and lens that measure the water thickness on the sample (details of the system are provided in the Section 4). Using the experimental arrangement shown in Figure 1b, the water thickness is evaluated through a lensing effect; a visual phenomenon caused by the $45^{{}^{\circ}}$ illumination angle and the concave shape of the water formed by the surface tension within the EM grid well. When the water reaches a thickness of approximately 10 μm, the camera reveals a distinct crescent-shaped pattern on the grid (Figure 1c).

### 2.2. Automatic Plunge-Freezing System

Another alternative to plunge-freezing methods is the use of automated systems, which are commercially available, such as the automatic systems by Termofisher, Leica, and Gatan. Some of these systems offer full control over sample preparation criteria [28], including sample environment and blotting parameters, which can influence the results.

In this study, we employed Vitrobot-IV (Thermofisher Scientific, Waltham, MA, USA). The schematic and details of this system can be accessed at www.thermofisher.com (accessed on 22 June 2024). This system facilitates sample vitrification within a controlled environment, e.g., humidity and temperature. The blotting parameters such as drain time, blotting time, and blotting force can also be set to ensure optimal sample vitrification.

**Figure 2:**
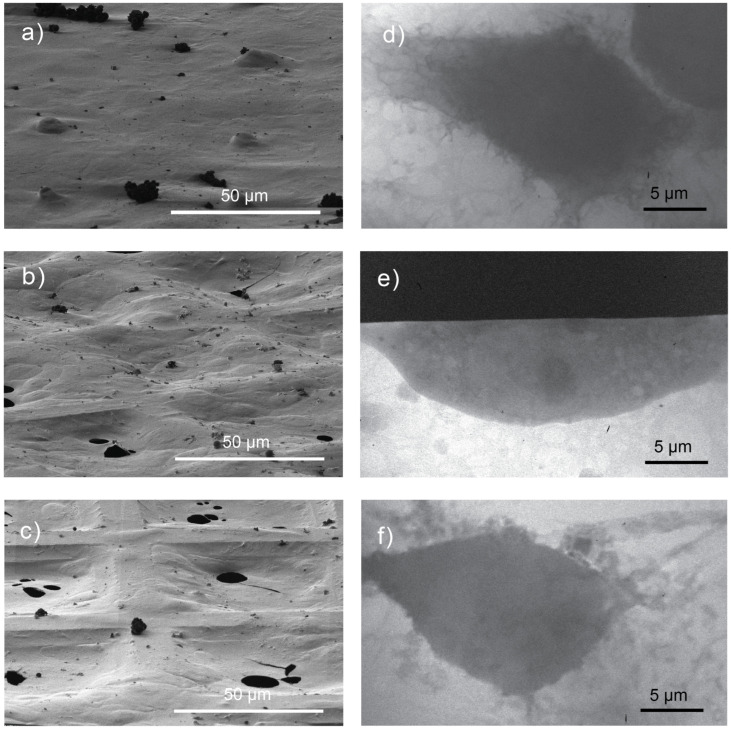
Images of adherent cells on the holed carbon layer of 300-mesh TEM grids captured under various plunge-freezing parameters. (**a**–**c**) Scanning electron microscopy (SEM) images of macrophages with blotting times of 1, 2, and 3 s, respectively. (**d**–**f**) LSXM macrographs of macrophages with blotting times of 1, 2, and 3 s, respectively.

Blotting is one of the critical parameters in the vitrification process of an automated system. By optimizing the blotting parameters, the water layer thickness on biological samples can be controlled. In this study, blotting time was found to greatly influence the thickness of the water around the cells, as demonstrated in Figure 2. As shown in Figure 2a–c, scanning electron microscopy (SEM) was used to image the surfaces of the TEM grids where the samples were seeded. The blotting times for these samples varied from 1 to 3 s. The SEM images show that with a blotting time of 1 s, the water thickness was significantly higher than the height of the TEM grid bars, as seen in Figure 2a. This water thickness decreased as the blotting time increased (Figure 2b,c).

The same batch of samples was also imaged using a laboratory soft X-ray microscope (LSXM) (Figure 2 right). The X-ray micrographs in Figure 2d–f indicate that both image quality and cell morphology were influenced by the blotting time. This is due to the great effect of the blotting time on the water thickness surrounding the cells, as previously mentioned. For the macrophage cells, a one-second blotting time (Figure 2d) resulted in poor contrast between the cell organelles and cytoplasm. This made organelle identification and classification challenging tasks. Despite this, the overall cell morphology appeared intact, with cells showing healthy adherence to the carbon layer on the TEM grid. In contrast, cells blotted for 3 s exhibited morphological alteration, including signs of membrane rupture (Figure 2f). A blotting time of 2 s was found to be optimal, as demonstrated in Figure 2e. At this blotting time, the natural contrast in the X-ray micrograph allowed for the observation of the cellular organelles, such as the nucleus and nucleolus. The cell also showed unnoticeable cellular morphological alterations.

### 2.3. ${\mathrm{MoO}}_{2}$ NP Uptake in Macrophages

**Figure 3:**
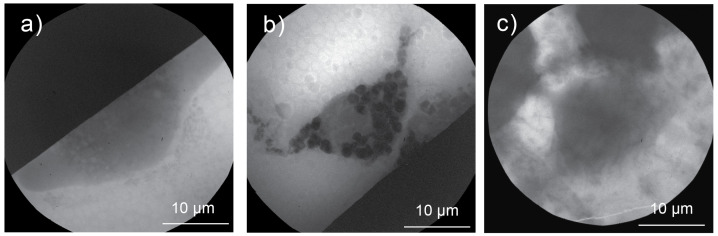
The effect of ice layer thickness on X-ray micrograph of macrophages exposed to ${\mathrm{MoO}}_{2}$ NPs. (**a**) Control cell. (**b**,**c**) Macrophages exposed to ${\mathrm{MoO}}_{2}$ NPs for 4 h. Samples in (**a**,**b**) were prepared using the optimized sample preparation, while (**c**) was prepared using inefficient blotting, resulting in a thick ice layer.

Figure 3 presents cryo X-ray micrographs of murine macrophages (RAW 264.7) exposed to ${\mathrm{MoO}}_{2}$ NPs under optimal and suboptimal sample preparation. Figure 3a depicts an X-ray micrograph of a control cell, which was blotted for 2 s during sample preparation. This blotting time was optimal for preserving cellular morphology, as also demonstrated in Figure 2e.

These cells retained their structural integrity, and the contrast provided was sufficient to distinguish cellular organelles, such as the nucleus and nucleolus. These observations suggest that optimal blotting conditions are critical to achieving the high-quality imaging of adherent cells.

The sample in Figure 3b was prepared using an optimized sample preparation protocol, enabling the achievement of a consistent ice layer thickness of 5–10 μm. In this sample, the presence of aggregated ${\mathrm{MoO}}_{2}$ NPs within the cytoplasm is clearly visible, highlighting the cellular uptake of the NPs. The observed morphological changes in the macrophages are likely attributable to the interactions between the ${\mathrm{MoO}}_{2}$ NPs and the cellular system. Such detailed observations are achievable in unstained cryo-samples using an LSXM, provided that proper sample preparation is carried out. In contrast, when the ice layer thickness is suboptimal (Figure 3c), the observation of ${\mathrm{MoO}}_{2}$ NPs within the cells becomes more challenging, complicating data interpretation. Additionally, a thick ice layer introduces difficulties in focusing during the imaging process, further compromising the quality and reliability of the observations.

### 2.4. ${\mathrm{MoO}}_{2}$ NP Uptake in Amoebas

**Figure 4:**
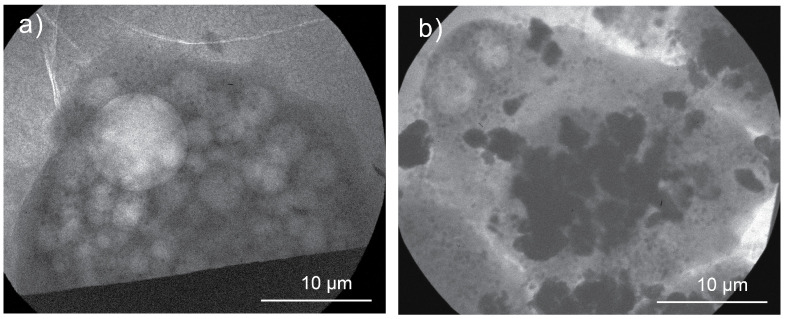
X-ray macrographs of amoebas exposed to ${\mathrm{MoO}}_{2}$ NPs. (**a**) Control cell. (**b**) Amoeba exposed to ${\mathrm{MoO}}_{2}$ NPs for 4 h. The samples were prepared with the optimized system.

Figure 4 shows X-ray micrographs of *Acanthamoeba castellanii* exposed to ${\mathrm{MoO}}_{2}$ NPs under the optimized parameters of the automated plunge-freezing system. Figure 4a presents an X-ray micrograph of a control cell blotted for 3 s during vitrification. This blotting time was found to be ideal for amoebas, likely due to their greater resilience compared to macrophages. The contrast achieved allows the observation of carbon-dense structures and contractile vesicles. In Figure 4b, in addition to the presence of carbon-dense structures and contractile vesicles, agglomerations of ${\mathrm{MoO}}_{2}$ NPs can be observed within the cells. These findings provide evidence of NP uptake by the amoebas.

## 3. Discussion

Achieving an optimal water layer thickness is difficult when preparing biological samples for an LSXM using cryofixation methods, such as plunge-freezing, to study bio–nano interactions. The ideal water thickness ranges between 5 and 10 μm, which is critical for maximizing soft X-ray transmission. Additionally, maintaining sufficient water is essential to keep the cells hydrated and structurally intact. In this paper, we propose two methods of achieving the optimal water thickness. The vitrification process has to be monitored to assess and evaluate the water layer thickness, and the sample environment and vitrification parameters must be controlled and optimized. The first approach can be performed in manual plunge-freezing using a monitoring system (Figure 1), while the second can be conducted on an automated setup (Figure 2). The representative results in Figure 3 and Figure 4 demonstrate the effectiveness of the presented protocol in facilitating the observation of cellular NP uptake.

The challenge with using LSXMs for cellular imaging is producing a sample with a minimal amount of water on the grid while keeping the cells hydrated. In the manual system (Figure 1a,b), a setup integrated with a camera monitoring system can be used to determine the thickness of water layer on the sample during vitrification. To obtain the optimal area containing the cell of interest using this method, the plunging process should begin when the crescent-shaped region covers approximately 20–40% of the grid area (Figure 1c). This ensures that the area adjacent to the crescent shape has a water thickness of less than 10 μm. This step typically takes 10–20 s after manual blotting. An earlier version of this setup has been used in early applications of LSXMs [1,17].

In plunge-freezing systems without a real-time mechanism to evaluate water layer thickness, excessive water removal during blotting can result in cellular dehydration or even cell death, as evidenced in Figure 2c,f. On the other hand, insufficient blotting might keep the cells in their hydrated and healthy (Figure 2a,d); however, samples with a thick ice layer may not completely vitrify [29]. Additionally, imaging a sample with a thick ice layer using an LSXM would not provide sufficient photon flux to observe cellular organelles and NPs. Thus, the blotting parameters have to adjusted to ensure the optimal ice layer thickness for the samples and particular imaging techniques. The optimal ice layer thickness of a biological sample after vitrification has to be less than 10 μm [2]. This is determined by the attenuation length of the X-ray radiation used for LSXMs [19,30].

The macrophages and amoebas exposed to ${\mathrm{MoO}}_{2}$ NPs (Figure 3 and Figure 4, respectively) serve as proof of concept that the systematic protocol presented here enables the observation of NP uptake across different cell types. The grid containing macrophages was blotted for 2 s, while the grid with amoebas was blotted for 3 s. These results demonstrate that the blotting parameters can be adjusted according to the cell type. From an imaging perspective, a longer blotting time is beneficial as it increases the X-ray transmission through the sample, thereby enhancing the contrast. However, it is crucial to fine-tune the blotting parameters cautiously to maintain a well-preserved biological sample while achieving the desired ice layer thickness. Vitrification parameters, including blotting time, must be optimized based on the sample’s specific characteristics and the requirements of the imaging technique employed.

A detailed workflow of the sample preparation protocol is provided in Appendix A. The first two steps of the workflow—plasma cleaning and cell seeding—are critical prerequisites for successful vitrification. Further details of these steps are elaborated in Steps I–X. A proper grid preparation or plasma discharge process (see the Section 4) is essential. Inadequate preparation can result in grids that are difficult to handle, exhibit high hydrophobicity, and have poor cellular adherence. These issues may compromise sample quality and necessitate discarding the sample at a later stage of preparation.

The cell concentration prior to the seeding process must be calculated based on the cell type, the surface area of the cell culture plate, and the volume of the media used. Ideally, 1–3 cells have to adhere to a 55 μm × 55 μm EM grid well (Step VI). In this study, the cell concentration was specifically adjusted for macrophages (RAW 264.7 murine macrophages) and amoebas (*Acanthamoeba castellanii*) using 3 mL of seeding medium per well in a 6-well plate. This cell concentration is also applicable for adherent cells with a size range of 20–50 μm [17,18].

With our presented protocol, we were able to produce images with high contrast using an LSXM by evaluating water thickness and modifying the plunge-freezing conditions in our manual plunge freezing system. Moreover, adjusting the blotting parameters of this automatic plunge freezing instrument was also proven to be the key factor in producing an optimum ice layer grid for SXM imaging. These are critical for obtaining high-quality images in the soft X-ray imaging of biological samples and for enabling the investigation of bio–nano interactions. A well-optimized ice layer enhances image contrast while minimizing artifacts. Notably, even with a lower-brightness X-ray source, this ice layer can yield image quality comparable to that achieved with a higher-brightness source, underscoring its importance in sample preparation and imaging workflows. The protocol could be further refined by exploring additional parameters, such as blotting force and drying time, and by expanding its application to a broader range of cell types.

## 4. Materials and Methods

### 4.1. Macrophage Sample

RAW 264.7 murine macrophages were used to evaluate the effectiveness of the protocol. The cells were cultured in a flask or petri dish with high-glucose DMEM (Sigma Aldrich, Solna, Sweden) until they were almost confluent, at which point they were ready to be seeded onto the grids. The cells were detached using TrypLE Express trypsin (Thermofisher Scientific, Waltham, MA, USA), counted, and exposed to 500 μg/mL ${\mathrm{MoO}}_{2}$ NPs before they were seeded on the grid. The optimal cell concentration for seeding the cells onto the grid in 6-well plates with a surface area of approximately 10 cm^2^ was $0.25\times 10^{6}$–$0.5\times 10^{6}$ cells/mL.

### 4.2. Amoeba Sample

*Acanthamoeba castellanii* was used to evaluate the protocol when a specific treatment was applied to the sample. In the beginning, the cells were adapted to PPYG medium containing 25 μg/mL amphotericin B, 50 μg/mL ampicillin, 100 units penicillin, and 100 μg/mL streptomycin (all from Sigma Aldrich, Solna, Sweden). The cells were then cultured in a flask until they almost reached confluence. They were then detached using PBS, counted, and exposed to 500 μg/mL ${\mathrm{MoO}}_{2}$ NPs before they were seeded on the grid.

### 4.3. ${\mathrm{MoO}}_{2}$ NP Synthesis and Characterization

The NPs were synthesized through a solvothermal method [31]. The precursor, ammonium heptamolybdate (3.6 mM), was dissolved in a solution of 54 mL of deionized (DI) water and 24 mL of ethanol absolute (EtOH, ≥99.820%). Subsequently, polyvinylpyrrolidone (0.29 mM) was added, and the mixture was stirred for 30 min. The synthesis process lasted for 18 h at 180 °C using a stainless-steel autoclave lined with Teflon. The NPs were collected by centrifugation and re-dispersed in DI water. MoO_2_ NPs exhibited a strong negative surface charge (ζ-potential: −39 mV). Primary MoO_2_ NPs (∼5 nm) formed clusters averaging 48 (±13) nm in the dry state (transmission electron microscopy, TEM) and 89 (±1) nm in dispersion (dynamic light scattering, DLS). The ζ-potential and hydrodynamic size were measured in triplicate of diluted solutions at neutral pH using a Zetasizer Nano ZS90 (Malvern Panalytical, Malvern, UK), with DLS values reported as volume-weighted averages. TEM (JEM-2100F, 200 kV, JEOL, Tokyo, Japan ) was used for morphology and size analysis of a 40 μL drop-casted sample (∼400 NPs/clusters) on copper grids, which were then dried at room temperature.

### 4.4. Grids for Biological Samples

Newly purchased grids are typically hydrophilic, but, over time, they accumulate charges and become hydrophobic. It is then necessary to use plasma cleaning (Pelco easiGlow, Tedpella, Redding, CA, USA), also commonly referred to as glow discharge, to restore the hydrophilicity of the grids. The plasma is created through the ionization of air under low vacuum pressure (<0.25 mBar). The plasma cleaning process is crucial because the hydrophobicity of the grids could negatively affect the experiment by reducing the adherence of the cells and grids with a charge, which can be challenging to handle. Plasma-treated grids also ensure that the liquid sample spreads evenly across the grid surface. In our laboratory-based SXM setup, the TEM grid with a holed carbon film was found to be more robust compared to lace carbon film and quantifoil carbon film. Given that our primary focus was working with adherent cells within the 20–50 μm size range [17,18,32], the utilization of 300- or 200-mesh grids with holed carbon film (S147A3, Agar Scientific, Essex, UK) offered a favorable combination of sturdiness and high X-ray transmission.

### 4.5. Manual Cryo Plunger

The manual plunge-freezing device used in this protocol is a home-built plunge-freezer. The schematic of the plunger is depicted in Figure 3b, which consists of a stand, bar clamp, plunging arm, cyrogen cup, and cryogen dewar. The stand is utilized to secure the plunging arm in place using a bar clamp. This bar clamp features a releasing cable system attached for plunging the plunging arm by pressing the release button. Additionally, a bar clamp is used to adjust the working height of the tweezers. The plunging arm is designed with a bump stop and grove connector. Finally, the plunger uses a cryogen dewar as a platform for the cryogen cup, liquid nitrogen, and grid storage (71166-10, Mitegen, Ithaca, NY, USA). In addition to the conventional components for plunge-freezing, our cryo plunger system integrates a camera monitoring system to assess the water thickness on the grids. We used a camera with a 1.3 megapixel CMOS sensor (IDS, Obersulm, Germany) and a lens system for this purpose. To provide illumination of the sample, a fiber-optic illuminator is utilized. The light source is strategically positioned at a $45^{{}^{\circ}}$ relative to the camera’s direction, creating a lensing effect on the water grids and preventing camera overexposure.

### 4.6. Automatic Cryo Plunger

In this study, we used an automatic system, Vitrobot-IV (Thermofisher Scientific, Waltham MA, USA). It was a good choice for this study because it could fully control the vitrification parameters, namely, temperature, humidity, blotting time, blotting offset, drain time, wait time, etc. Vitrification using Vitrobot has been well established for cryo-TEM [33]. In this study, we adjusted the cryo-TEM procedure to meet the criteria for the sample requirements in the LSXM. The full Vitrobot-IV specifications and manual can be accessed at: https://www.thermofisher.cn/cn/en/home/electron-microscopy/products/sample-preparation-equipment-em/vitrobot/instruments/vitrobot-mark-iv.html (accessed on 22 June 2024).

### 4.7. Microscopy

X-ray images were acquired using an LSXM as previously described in Refs. [1,32]. This microscope operates using an X-ray source generated from nitrogen plasma, which is produced by focusing a 1064 nm diode-pumped Nd:YAG slab laser (Fraunhofer ILT, Aachen, Germany) onto a 25 μm liquid nitrogen jet. All X-ray images were captured with 800× magnification and using a 30-s exposure time. Scanning electron microscopy (SEM) images were acquired using an Aquilos 2 Cryo FIB (Thermofisher Scientific, Waltham, MA, USA). The images were captured with 1200× magnification using a 30 kV acceleration voltage.

## 5. Conclusions

We developed a systematic protocol for preparing biological samples for LSXMs. This method allows the attainment of a 5–10 μm thick ice layer on the samples, which is a critical requirement for LSXMs. A controllable ice thickness allows higher X-ray transmission through the sample, enabling the observation of cell organelles, NP uptake, and localization. The efficacy of the method was successfully demonstrated with both macrophages and amoebas. Overall, these findings suggest that this protocol shows significant promise for addressing the challenges related to the low X-ray transmission in cellular imaging using LSXMs.

## Data Availability

The datasets used and/or analyzed during the current study are available from the corresponding author upon reasonable request.

## Abbreviations

The following abbreviations are used in this manuscript:

SXM	Soft X-ray microscopy
LSXM	Laboratory soft X-ray microscope
EM	Electron microscopy
NPs	Nanoparticles

## Appendix A. Experimental Procedures

### Appendix A.1. First Phase: Grid Preparation

- Place the grid onto a microscope slide. If preparing multiple grids, cover the surface of the slide with Parafilm to facilitate transfer of the grids after the plasma cleaning process.
- Insert the microscope slide containing the grids into a glow discharge chamber (Pelco easiGlow, Ted Pella, Redding, CA, USA) for plasma cleaning. Clean the grids at a current of 15 mA under a pressure of 0.25–0.1 mBar for 1–2 min. Position the grids near the center of the chamber to ensure the uniform exposure of the carbon layer to the plasma.
- Transfer the grids from the microscope slide to a 6 well-plate. Ensure that the carbon side of each grid is facing downward, and place up to four grids per well. This step must be performed in a fume hood to avoid contamination.

### Appendix A.2. Second Phase: Cell Seeding

IV. Remove the medium from the cultured cells, wash 1× with PBS (pH7), and detach the cell with TrypLE.
V. Resuspend the detached cells in high-glucose DMEM to neutralize the TrypLE. Mix the suspension gently to avoid cell clumping.
VI. Prepare cell concentration. We used an automatic cell counter (Invitrogen Countess II, Thermo Fisher Scientific, Waltham, MA, USA). Ensure that the percentage of dead cells is below 20% before proceeding. The optimal cell concentration for seeding onto the grids in the 6-well plate (surface area: 9.6 cm^2^) (Thermo Fisher Scientific, Waltham, MA, USA) is $0.25\times 10^{6}$–$0.8\times 10^{6}$ cells/mL.
VII. Apply experimental treatment to the cells at this step. For example, in this study, macrophage and amoebas were exposed to NPs.
VIII. Transfer the prepared cell suspension from Step VII onto the grids prepared in Step III. Carefully cover the grid with approximately 100–200 μL of the cell suspension before filling the remaining 6-well plate.
IX. Incubate the cells in a humidified incubator for 1–2 h to allow the cells to adhere to the carbon film on the grids.
X. Check the cell adherence and confluence. Aim for 1–3 cells per EM grid well.

### Appendix A.3. Third Phase: Sample Vitrification

XI. Remove the supernatant from the 6-well plate and resuspend the grids with PBS.
XII. Transfer the grids to another 6-well plate pre-filled with PBS. Repeat this process 1–2 times before proceeding to then next step.
XIII. Evaluate the grids using a light microscope. Assess the cell concentration, morphology, and overall health, ensuring the cells are adequately spread out and healthy. Moreover, the shape of the grid is also assessed during this step.
XIV. Begin the cooling process by filling the cryogen dewar with liquid nitrogen.
XV. Allow the cryogen cup to reach a temperature of approximately −170 °C. This process takes about 15 min.
XVI. Once the cryogen cup reaches the desired temperature, remove any residual liquid nitrogen before initiating the ethane condensation process.
XVII. Fill the cryogen cup with liquid ethane, ensuring that the liquid ethane level reaches 80–90% of the cup’s capacity. Continuously monitor and refill both the liquid nitrogen and ethane levels throughout the vitrification process, as their levels decrease over time. This step must be conducted in a well-ventilated, spark-free environment to prevent safety hazards.

### Appendix A.4. Manual Plunge-Freezing

XVIII. a. Pick up the grid gently by gripping it on the rim with an inverted tweezer modified for manual plunge-freezers. Ensure the grid is securely mounted on the tweezers by tapping them gently. Once secure, attach the tweezers to the plunging arm.
XIX. a. Raise the plunging arm to its uppermost position until the release button can be activated. In this position, the grid is in the camera focus for water thickness evaluation and adjustment. As previously mentioned, illumination light positioned at a 45-degree angle relative to the camera’s axis helps visualize the water thickness on the grid. Areas with more water (wet grid) appear darker than those with less water.
XX. a. Blot the grid using blotting paper to remove excess water. Blot the grid from the back side (carbon film side). Start the blotting process by removing the excess water near the tweezer tips. Remove the remaining excess water from the grid by moving the blotting paper around the grid’s rim, while monitoring the water thickness on the camera.
XXI. a. Continue blotting until a crescent-shaped region appears on the camera. This visual effect results from the 45-degree illumination angle and the concave water meniscus within the grid well. Stop the blotting process once this crescent-shaped area covers approximately 20–40% of the grid’s surface.
XXII. a. Initiate the plunging process by pressing the release button.

### Appendix A.5. Automatic Plunge Freezing (Vitrobot-IV)

The setup and manual for Vitrobot-IV can be found at: https://assets.thermofisher.com/TFS-Assets/MSD/manuals/vitrobot-mk-iv-user-manual-pn103261.pdf (accessed on 22 June 2024):

XVIII. b. Using the specialized Vitrobot-IV tweezers, gently pick up the grid by its rim. These tweezers feature a clamp ring locking system, which can be opened or closed by sliding the ring along the grooves. As in the manual’s procedure, ensure the grid is securely mounted by tapping the tweezers lightly before attaching them to the plunging arm.
XIX. b. Secure the tweezers onto the plunging arm, and close the chamber.
XX. b. Set the plunge-freezing parameters via the Vitrobot-IV user interface. For macrophage samples, set the humidity to 80–90%, the temperature to 16 °C, the blotting force and draining time to 0, and the blotting time to 2 s. For amoeba samples, the humidity, temperature, blotting force, and draining time are set to the same values as for the macrophages but the blotting time is set to 3 s. Both samples undergo a single blotting cycle under these conditions.
XXI. b. Allow 10–20 s for the chamber to reach the desired humidity before proceeding with the plunge-freezing process.
XXII. b. Initiate the grid blotting and plunging process by pressing “Continue” on the user interface or using the foot pedal. After this, the grid is immediately blotted and plunged into liquid ethane.

### Appendix A.6. Fourth Phase: Storing the Sample

XXIII. Detach the tweezers from the plunging arm while keeping the grid submerged in liquid ethane.
XXIV. Transfer the grid from the liquid ethane to liquid nitrogen and place it in grid storage (Mitegen, Ithaca, NY, USA). Avoid exposing the grid to atmospheric moisture during this transfer to prevent the formation of ice artifacts on the samples.
XXV. Remove any condensed water from the tweezers. Repeat Steps XVIII–XIV for subsequent grids. Alternatively, use a second pair of tweezers to process new grids while the first pair is being heated and dried.
XXVI. Close the grid storage and transfer it to a 30 L cryo dewar (Mitegen, Ithaca, NY, USA). Keep the grids submerged in liquid nitrogen within the dewar until they are ready for SXM imaging. Ensure all equipment used for sample transfer is pre-cooled to prevent thermal shock.
